# The Methods of Microscopical Examination and of Artificial Culture of Bacteria

**Published:** 1886-05

**Authors:** George C. Hummel

**Affiliations:** Savannah, Ga.


### THE METHODS OF MICROSCOPICAL EXAMINATION AND OF ARTIFICIAL CULTURE OF BACTERIA.

READ BEFORE THE MEDICAL ASSOCIATION OF GEORGIA, AT SAVANNAH, APRIL 15TH, 1885.

BY GEORGE C. HUMMEL, M. D., SAVANNAH, GA.

(CONCLUDED.)

If micro-organisms are detected with the aid of the microscope, the artificial culture is absolutely necessary for the further study of their life-history.

To simply follow up the morphological changes which they undergo during their development, it is sufficient to observe them directly under the microscope. For this purpose a minute portion of the material containing the bacteria and a drop of suitable nourishing medium is placed on a thin cover-glass, and this is fastened by parafine or wax, face downwards on a slide, which has in its center a concave pit, making a so-called culture-cell. On a heated stage the thus sown bacteria may be observed, even with the highest powers, or the specimen may be placed in an incubator at a desired temperature and examined from time to time. Yet these observations cannot be carried on for any length of time, as the culture-cell is not entirely secure against impurities from the outside.

For a more thorough study of the bacteria, which arouse our interest, further and more extensive cultivation is necessary.

As receptacles for some of these cultures, testubes, provided with a long and dense cotton plug to prevent the accidental entering of other germs, are used. For others, again, small glass dishes, one-fourth of an inch high and two inches in diameter, fitting into cylindrical glasses about six inches high and two and a half inches in diameter. A strip of brass sheeting, half an inch wide, and curved into the shape of an L, is used for lifting the glass dish in and out. The cylinder is closed, like the testube, by a tightly fitting cotton plug. For other varieties a number of glass slides or plates are used and arranged on a small glass or metal stand. They are kept under a bell-glass or in a suitable glass dish. A piece of blotting paper, soaked with distilled water, for supplying the necessary moisture, is placed under the stand. It is also advisable to glue a strip of blotting-paper in the bell, as in consequence of the evaporation going on, drops of water will collect in the bell, and might fall upon the cultures and thus spoil them.

All these receptacles are sterilized, as before described, and provided with the different nourishing materials, the careful composition of which is of the greatest importance. Not every nourishing medium will suit every micro-organism. We have to consider if they thrive better under alkaline or acid reaction. Some prefer a neutral condition of the nourishing material. For cultivation of moulds, a culture ground which contains only a small percentage of water and shows a decided acid reaction, is preferable. For yeasts, the most favorable conditions are met by fluids of well pronounced, yet not excessive acid reaction, and such which contain an abundance of sugar. Bacteria prefer neutral, not too concentrated media. Natural nourishing materials suitable for moulds are decoctions of dried prunes or raisins, infusions of the fresh fæces of herbivoræ or decoctions of yeast with addition of sugar and slices of bread, steeped in the decoctions named. If these substances do not react sufficiently acid, tartaric acid (2 to 5 per ct.) or phosphoric acid (½ to 1 per ct.) may be added. Yeasts thrive well in decoctions of malt, in grape juice, beer, or the above-named decoction with addition of grape sugar. For bacteria use urine, blood serum, hay infusions and meat infusions, which react neutrally or are only very slightly alkaline.

Nægeli recommends for the schizomycetes the following fluid media: (1.) Water 100 grammes, ammonium tartrate 1 grm, potassium phosphate 1 grm., magnesium sulphate 0.02, calcium chloride 0.01. (2.) Water 100.00, albumen 1.00, potassium phosphate 0.2, magnesium sulphate 0.04, calcium chloride 0.02. (3) Water 100.00, cane-sugar 3.00, ammonium tartrate 1.00, mineral substance as in 2.

But of all the nourishing material the solid culture media have gained of late the greatest importance. Sliced potatoes, turnips, beets, and other vegetables do very well for many purposes, but the above-named nourishing fluids mixed with gelatine or agar-agar solution are the most preferable ones. Solidified blood serum, the preparation of which I shall describe later, is an excellent nourishing medium.

All these nourishing materials have to be sterilized previous to use. Potatoes, turnips, etc., are washed with a solution of corrosive sublimate (1:2000), rinsed off with distilled water and exposed to a heat of 100° in a steam apparatus for about thirty minutes. After cooling they are cut in two by previously heated knives, and, if perfect security against impurities is desired, placed in cylindrical well-plugged glasses, and again exposed for ten minutes to steam of 100°. In most cases it will suffice to keep them under previously sterilized glasses.

The gelatine-meat infusion is prepared, according to Koch, in the following manner: Pour one liter of distilled water over onehalf kilo of fresh beef, finely chopped and freed from all connective tissue and fat. Set aside for twenty-four hours in cold, for twelve hours in warm weather; strain and press forcibly through muslin; keep in steam of 100° C. for half an hour, filter through filtering paper; add 15 grms. peptone, 7½ grms. chloride of sodium; dissolve and filter; add enough distilled water to make 1½ liter; sterilize for ten minutes and cool. Then add 75 grms. of finest gelatine cut in small strips, dissolve in water bath and add a few drops of saturated watery solution of carbonate of sodium, until the solution shows besides the acid reaction (on account of the phosphoric acid present) a pretty decided alkaline reaction. Subject again to steam of 100° C. for ten minutes, and filter while hot. If the filtered fluid is cloudy the filtering must be repeated until quite a clear fluid is attained. Bring again in steam apparatus for ten minutes (by no means longer, as the gelatine might lose the power of solidification if heated longer) and fill the previously sterilized testubes to about one-third their contents. The filled testubes must be exposed to steam of 100° C. for four days in succession, five minutes each day. If they then keep for several days without getting turbid, they are ready for use. The gelatine will become fluid in a temperature of 20° C. The agar-agar solution, which is prepared exactly as the gelatine solution, with the exception that instead of 5 per cent. gelatine, only 1 or 2 per cent. agar-agar is used, keeps solid in a temperature up to 40° C., and is therefore preferable in a warm climate. A great drawback to its use is its turbidness.

Blood serum is thus prepared (after Koch): The throat of the animal which is to be slaughtered is carefully washed off with a solution of corrosive sublimate in the proportion of 1:2000, which is rinsed off again with alcohol. The blood is gathered in narrow and high cylindrical glasses, previously washed with solution of corrosive sublimate (1:2000) and rinsed with alcohol, and set aside for 24:30 hours until the serum separates. This is drawn off with a sterile pipette into sterile large testubes, which are again set aside to let the blood corpuscles that may have been accidentally stirred up settle. The perfectly clear serum is then poured into sterile small testubes to about one-third of their contents. These are exposed for six successive days to a heat of 58° C.=134° F. in a common incubator one-half an hour each time.

On the seventh day they are kept in the incubator in a strongly inclined position, to give as much surface as posible, at a heat of 65° C.=150° F. for one-half to one hour, until they are solidified. They are then set aside for a few days, eventually at a heat of 30–35° C.=85–95° F. to make sure that no changes, caused by impurities, take place.

All these operations may seem tedious, but we cannot be too scrupulous in these matters. Ill success is sure to follow negligence. The work of weeks may possibly be spoiled by the slightest omission of the necessary precautions.

Special methods are required if the sowing material is not pure, that is, if, besides the desired one, other micro-organisms are present.

Different methods have been proposed by Pasteur, Klebs, Brefeld, Nægeli, Buchner and others, but after having worked with all of them, the method of Koch seems to me to be the only one which offers a positive certainty to obtain pure cultures. I refer to the solid culture grounds. They had been used before, but Koch was the first to propose them for the purpose of isolating pure cultures.

While in fluid nourishing media the sowed and the accidentally entered micro-organisms get mixed, so that we can hardly recognize those which develop slow among the larger number of those which develop faster, on the solid culture ground each colony will be isolated and therefore easily recognized. If we inoculate a certain kind of bacterium in many different places upon a solid culture ground, small colonies will form around each spot of inoculation, which may very soon be observed microscopically; they have generally a characteristical color, consistence and shape. If other bacteria settle upon the same culture ground, they will form colonies for themselves, which mostly do not mix up with the inoculated ones, and which differ materially from them in the three properties named. But should they fall upon an inoculated colony, the appearance of the same will be changed, and we may easily discover by a miscroscopical examination, if any impurities are present at a spot, from which we desire to further inoculate. Only those colonies which are found to be absolutely pure are used, and in this certainty lies the advantage of the solid culture medium over the fluid one. If strange bacteria have entered into the latter, they spread with rapidity over the whole medium, and it is mere accident if they are not inoculated together with the desired one. The previous microscopical examination is certainly not of corresponding advantage, because if it proves the presence of impurities it is too late to yet obtain a pure culture. Further dilution has to be proceeded with, and by that manipulation the whole culture is endangered. On the contrary, in the solid culture media this scrupulous carefulness is not so essential, for, as I said before, the spot from which the sewing material is to be taken is always selected by microscropical examination.

For these solid culture grounds the previously described gelatine or agar-agar meat solutions are very suitable. They are either spread on common glass slides, in the before-named small glass dishes, or on glass plates.

The slides are prepared in this way: The contents of a testubeful of sterile gelatine solution is liquified in the waterbath, sucked up by a pipette, previously sterilized with the aid of heat, and poured out in small strips upon the slides. As soon as the gelatine has solidified, the sowing material is taken up by a sterile platinum wire and drawn across the gelatine in three or four transverse lines. These slides are kept upon suitable glass or metal stands under bell-glasses, prepared in the same manner as those used in potatoe cultures, or in suitable glass dishes.

If perfect security against impurities from the air is desired, the second method is used. The liquified gelatine is poured in small glass dishes which fit in well-stoppered cylinder-glasses. The whole apparatus is then exposed to steam of 100° C. for ten minutes. After cooling the sowing material is inoculated in the same manner as on the slides. If the glass is closed again quickly, there is a good chance that no germs from the air will enter.

Both of these cultures may be observed directly under the microscope with low powers (100–200). The growing colonies along the lines can be easily observed and the peculiar form of each recognized.

If we desire to isolate one special micro-organism from material containing large numbers of different bacteria, the third method is used. This is a combination of the ones just described with the method of dilution as proposed by Naegeli and Lister. A trace of the sowing material is shaken up with the liquified contents of a testube of gelatine solution, and this mixture is poured upon a sterile glass plate. After cooling it is placed upon suitable stands under bell-glasses, or in dishes as before.

If a still further dilution is desired, shake a drop of the sowing material with a testubeful of some sterile, indifferent solution, as a ¾ per cent. solution of sodium chloride, and of this mixture take again a few drops to a tube of gelatine and proceed as before. All over the plate the colonies will develop in a short time. The slides as well as the glass plates have to be carefully leveled or the liquified gelatine will flow over at some point.

After having found, with the aid of the microscope and the staining process, a perfectly pure colony, a trace of it is inoculated with a sterile platinum wire into a sterile testube, containing the most favorable nourishing medium. The cotton plug ought to be raised and replaced as qnickly as possible, as there is always danger that some germ may fall out of the air upon the nourishing medium and thus spoil the culture. Whenever we want to be certain, we ought to inoculate a number of testubes, and some out of the number will prove pure, as the danger from impurities out of the air is not as great as from impurities clinging to the glass, cotton and other articles. Most cultures will keep six to eight weeks, many considerably longer. After that time fresh culture tubes should be inoculated, and thus the culture may be kept up *ad infinitum*.

It is certainly advisable, before trying to experiment with new bacteria, to learn the method thoroughly by cultivating well-known varieties. I recommend for that purpose the micrococcus prodigiosus upon potatoes and in gelatine solution, the baccillus anthracis upon potatoes in gelatine solution and blood serum at different temperatures and through many generations.

If everybody who experiments with bacteria, but especially with the isolation of pathogenic ones, would test his knowledge with some well-known forms, many unripe publications, which can only injure this comparatively new branch of pathology, would be left unwritten.

If we have succeeded in obtaining a pure culture of a bacterium, its biology ought to be studied. We have to find out which nourishing medium and what temperature are the most favorable ones to its growth. We have to see if it is possible to cause fermentation by it, also if oxygen is essential to its growth, and at last we have to find out if it is pathogenic or septic. Animals, which are susceptible to infectious diseases, as mice, rabbits, guinea pigs or monkeys, ought to be vaccinated with smaller or larger doses and the effect carefully watched. Subcutaneous and injections into the internal organs ought also to be tried. If the animals die, we have to try to obtain again pure cultures out of their blood and see if these are in every particular the same as the original ones. All these trials ought to be repeated again and again.

And principally we ought to experiment under which condition they will perish and which disinfecting media will affect them most.

I hope that I have succeeded in giving you a clear idea of the microscopical examination and the artificial culture of bacteria, and that I may be able perhaps at a future meeting to make you acquainted with some micro-organisms peculiar to this climate.

